# GLP-1 Analogue Liraglutide Attenuates Mutant Huntingtin-Induced Neurotoxicity by Restoration of Neuronal Insulin Signaling

**DOI:** 10.3390/ijms19092505

**Published:** 2018-08-24

**Authors:** Ching-Chi Chang, Tzu-Chin Lin, Hsiao-Li Ho, Chien-Yin Kuo, Hsin-Hua Li, Tatiana A. Korolenko, Wei-Jen Chen, Te-Jen Lai, Ying-Jui Ho, Chih-Li Lin

**Affiliations:** 1 Institute of Medicine, Chung Shan Medical University, Taichung 40201, Taiwan; fmaj7@seed.net.tw (C.-C.C.); lily09102352000@gmail.com (H.-L.H.); a9704119@hotmail.com (C.-Y.K.); vivid529@hotmail.com (H.-H.L.); ltj3123@ms2.hinet.net (T.-J.L.); 2 Department of Psychiatry, Chung Shan Medical University Hospital, Taichung 40201, Taiwan; jamaalbest@gmail.com; 3 Federal State Budgetary Scientific Institution, Scientific Research Institute of Physiology and Basic Medicine, Novosibirsk 630117, Russia; t.a.korolenko@physiol.ru; 4 Department of Biomedical Sciences, Chung Shan Medical University, Taichung 40201, Taiwan; cwj519@csmu.edu.tw; 5 Department of Psychology, Chung Shan Medical University, Taichung 40201, Taiwan; 6 Department of Medical Research, Chung Shan Medical University Hospital, Taichung 40201, Taiwan

**Keywords:** autophagy, huntingtin, insulin signaling, liraglutide, oxidative stress

## Abstract

Huntington’s disease (HD) is a progressive and fatal neurodegenerative disease caused by CAG repeat expansion in the coding region of huntingtin (HTT) protein. The accumulation of mutant HTT (mHTT) contributes to neurotoxicity by causing autophagy defects and oxidative stress that ultimately lead to neuronal death. Interestingly, epidemiologic studies have demonstrated that the prevalence of type-2 diabetes, a metabolic disease mainly caused by defective insulin signaling, is higher in patients with HD than in healthy controls. Although the precise mechanisms of mHTT-mediated toxicity remain unclear, the blockade of brain insulin signaling may initiate or exacerbate mHTT-induced neurodegeneration. In this study, we used an in vitro HD model to investigate whether neuronal insulin signaling is involved in mHTT-mediated neurotoxicity. Our results demonstrated that mHTT overexpression significantly impairs insulin signaling and causes apoptosis in neuronal cells. However, treatment with liraglutide, a GLP-1 analogue, markedly restores insulin sensitivity and enhances cell viability. This neuroprotective effect may be attributed to the contribution of the upregulated expression of genes associated with endogenous antioxidant pathways to oxidative stress reduction. In addition, liraglutide stimulates autophagy through AMPK activation, which attenuates the accumulation of HTT aggregates within neuronal cells. Our findings collectively suggest that liraglutide can rescue impaired insulin signaling caused by mHTT and that GLP-1 may potentially reduce mHTT-induced neurotoxicity in the pathogenesis of HD.

## 1. Introduction

Huntington’s disease (HD) is a progressive neurodegenerative disease that affects motor and cognitive functions and results in complete physical and mental deterioration. It is a fatal genetic disorder that currently has no known cure or even treatment. The primary pathology of HD is caused by a mutation in the gene that encodes for the huntingtin (HTT) protein. Although the exact function of HTT is unknown, it may participate in neuronal survival and in various neuronal functions, including synaptic signaling, vesicle trafficking, and microtubule binding, and may also protect against programmed cell death [1]. Mutant HTT (mHTT) protein is a key factor in the induction of HD. Exon 1 of the normal HTT gene contains 10–35 CAG trinucleotide repeats, whereas that of mHTT may carry 36 or more expanded repeats and is associated with a pronounced HD phenotype [2]. In particular, the number of repeats is roughly correlated with disease severity and age at disease onset [3]. This association indicates that the size of the CAG repeat plays a critical role in the pathogenesis of HD. Although the exact neurotoxic mechanisms involved in mHTT-induced neurodegeneration have not yet been unraveled, the accumulation of mHTT affects various cellular functions and causes neurotoxicity [4]. mHTT itself tends to aggregate within the cytosol [5]. mHTT aggregates have been implicated as the major culprits of neurodegeneration, as well as of the initiation of mitochondrial dysfunction and the production of reactive oxygen species (ROS) that could then eventually lead to synaptic dysfunction and apoptosis [6]. Moreover, mHTT aggregates cause the impairment of the autophagy-mediated degradation of misfolded proteins, thereby facilitating the initiation of mHTT aggregate accumulation [7].

mHTT is the direct mediator of cell toxicity and death. In fact, the presence of mHTT aggregates contributes to superoxide anion formation and related oxidative stress in neurons. Interestingly, the prevalence of diabetes among patients with HD is higher than that among matched healthy controls, implying that HD progression may lead to defects in insulin signaling [8]. Furthermore, large mutant CAG repeat sizes are associated with decreased insulin sensitivity [9]. This relationship suggests that polyQ expansion in mHTT may contribute to impaired insulin signal transduction [10]. A large body of evidence suggests that insulin resistance may cause or promote neurodegeneration [11]. Defective insulin signaling in the brain is associated with decreased neuronal survival and cognitive function [12]. Therefore, the sensitization of neuronal insulin signaling may be useful in the treatment of diseases characterized by protein aggregation, such as HD [13]. For example, the direct administration of insulin alleviates mHTT-induced metabolic dysfunction [14], mitochondrial dysfunction, and ROS production [15]. Furthermore, we have previously reported that treatment with the dipeptidyl peptidase-4 inhibitor linagliptin may increase the levels of neuronal GLP-1 and provide neuroprotection in human SK-N-MC neuronal cells. The neuroprotective effect of GLP-1 may be associated with a mechanism that is dependent on neuronal insulin signaling [16]. Previous studies have demonstrated that exendin-4, a GLP-1 analogue, can improve motor function and extend the survival time of mHTT-expressing mice, thus suggesting that GLP-1 may be a promising agent for therapeutic intervention in HD [17]. However, the mechanism underlying these effects remains largely unknown. In the present study, we demonstrate that the blockade of neuronal insulin signaling may be a mechanism that underlies mHTT-induced neurotoxicity. We also show that liraglutide, a human GLP-1 analog with 97% homology to native GLP-1, can contribute to the alleviation of mHTT-induced neurotoxicity and protect SK-N-MC cells by restoring impaired insulin signaling.

## 2. Results

### 2.1. Q74-mHTT Overexpression Causes Significant Neuronal SK-N-MC Cell Death

**Figure 1:**
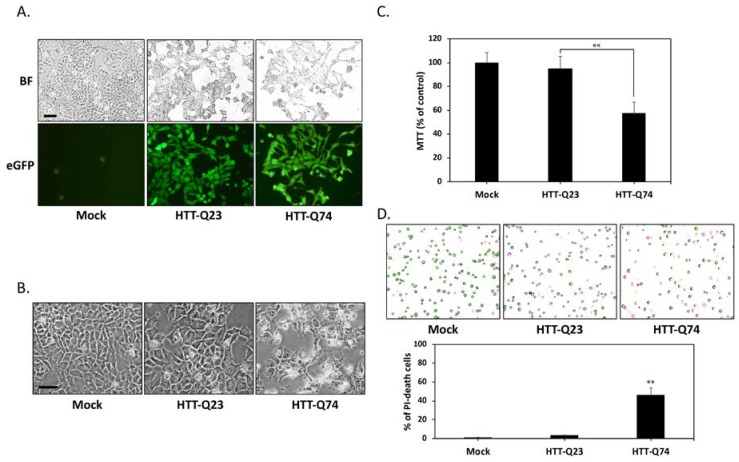
Q74-mHTT overexpression induces the apoptosis of human neuronal SK-N-MC cells. (**A**) SK-N-MC cells were transfected for 24 h with the empty vector (mock), normal polyQ HTT (pEGFP-Q23 HTT), or polyQ-expanded mHTT (pEGFP-Q74 HTT). The presence of green fluorescent reporter eGFP signals indicated the successful delivery and expression of HTT-Q23 or HTT-Q74 plasmids. BF, bright field. Scale bar represents 20 μm; (**B**) Phase-contrast microscopy images of cells taken after 48 h of transfection. In contrast to HTT-Q23-overexpressing cells, mock-transduced cells did not show any morphological characteristics of cytotoxicity or cell death. However, cell death markedly increased among HTT-Q74-overexpressing cells. Scale bar represents 20 μm; (**C**) Colorimetric MTT assay results for cell proliferation indicated that after 48 h of transfection, cell death reached 43% in the HTT-Q74-overexpressing group relative to that in the mock- or normal HTT-transduced groups; (**D**) AO–PI double staining assay results obtained with an automated dual fluorescence cell counter. AO conferred green fluorescence to live cells (green circles), and PI conferred red fluorescence to dead cells (red circles). The numbers of apoptotic cells were quantified by averaging the cell counts of five independent samples. At least three independent experiments were performed, and values are presented as mean ± SEM. Significant difference was determined by using the multiple comparisons of Dunnett’s posthoc test for ** *p* < 0.01 compared with mock groups.

To create a cellular model of mHTT-induced neurotoxicity, we first transiently transfected HTT expression vectors into SK-N-MC human neuronal cells. The cells were transfected with plasmids overexpressing normal HTT (HTT-Q23) or mHTT (HTT-Q74). At 24 h posttransfection, transfection efficiency was assessed on the basis of GFP expression under fluorescence microscopy. Figure 1A shows that the GFP gene was markedly overexpressed. This expression pattern suggested the successful delivery and expression of HTT-Q23 or HTT-Q74 plasmid into cells. Given that the accumulation of mHTT is essential for its neurotoxic effects, we evaluated cytotoxicity at 48 h post transfection. Our results showed that that overexpression of normal HTT did not induce significant toxicity. By contrast, mHTT markedly induced cell death (Figure 1B). Accordingly, the results of our MTT assays that cell death rates of the mHTT-overexpressing group reached 43% (Figure 1C). Data obtained were further confirmed by the results of the AO–PI double staining assay, which was performed by using an automated dual fluorescence cell counter. As shown in Figure 1D, the AO–PI assays revealed that compared with that of mock- or normal HTT groups, the number of viable cells of the mHTT-overexpressing groups decreased after 48 h of transfection. Our results collectively demonstrate that mHTT overexpression can cause significant cytotoxicity in human SK-N-MC neuronal cells.

### 2.2. Q74-mHTT Overexpression Stimulates Apoptosis and Blocks Insulin Signaling

**Figure 2:**
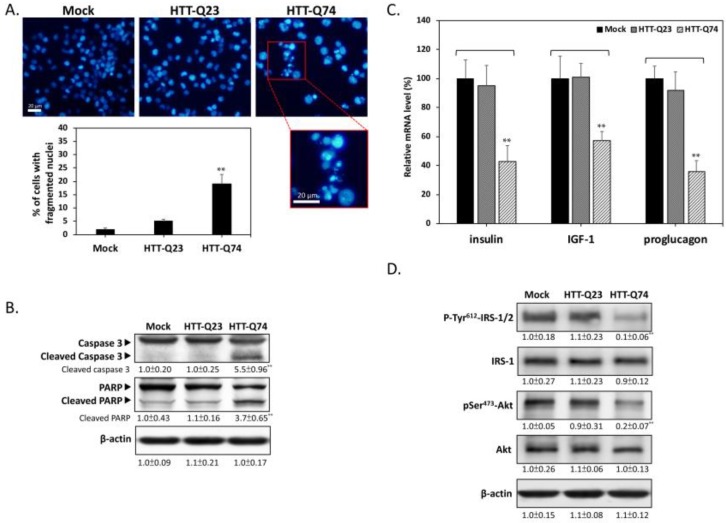
Q74-mHTT overexpression induces apoptosis and insulin signaling blockade in SK-N-MC neuronal cells. (**A**) Nuclear fragmentation markedly increased in HTT-Q74-overexpressing cells compared with that in mock- or HTT-Q23 transduced groups after 48 h of transfection. Results were determined on the basis of fragmented nuclear morphology through DAPI fluorescence; (**B**) Results of Western blot analysis demonstrated that HTT-Q74 overexpression stimulates caspase 3 and PARP activation; (**C**) Real-time qPCR was used to measure the mRNA levels of insulin-related genes, including insulin, IGF-1, and proglucagon; (**D**) Immunoblotting revealed that the phosphorylation of Tyr^612^-IRS-1/2 and Ser^473^-Akt was upregulated when cells were transfected with HTT-Q74 for 48 h. All data were collected from at least three independent experiments, and values are presented as mean ± SEM. Significant difference was determined through multiple comparisons with Dunnett’s posthoc test for ** *p* < 0.01 compared with mock groups. Scale bar represents 20 μm.

To determine the mode of cell death that is induced by mHTT, we used DAPI nuclear staining to investigate the occurrence of nuclear condensation and fragmentation. As shown in Figure 2A, nuclear fragmentation was significantly enhanced in the HTT-Q74-overexpressing group compared with that in the mock- or HTT-Q23 transduced groups after 48 h of transfection. This result was also confirmed through Western blot analysis, which showed that HTT-Q74 increased the cleavage of caspase 3 and PARP, which are two typical apoptosis markers (Figure 2B). The expression levels of insulin and IGF-1 are downregulated in brain neurons derived from mHTT knock-in mice [15]. To determine the effects of HTT on the expression of genes related to insulin signaling, we performed relative expression qPCR assays to measure the levels of mRNA transcripts in SK-N-MC cells. As shown in Figure 2C, HTT-Q74 overexpression in SK-N-MC cells strongly suppressed the mRNA levels of insulin, IGF-1, and proglucagon (the pro-hormonal precursor mRNA of GLP-1) over a 48 h period. However, no significant changes were found in the expression of these mRNA transcripts in the HTT-Q23-overexpressing group compared with that in the mock control, indicating that mHTT overexpression may downregulate insulin/IGF-related signaling in neuronal cells. To further elucidate whether mHTT overexpression interferes with insulin signaling, we conducted Western blot analysis to detect the levels of the phosphorylation of insulin receptor substrate-1/2 (IRS-1/2) at residue Tyr^612^, which is a hallmark that positively regulates insulin signaling. As shown in Figure 2D, 48 h of HTT-Q74 overexpression decreased Tyr^612^ IRS-1/2 phosphorylation [18]. Accordingly, the phosphorylation of the insulin downstream target Ser^473^ Akt decreased in the HTT-Q74-overexpressing group relative to that in mock- or HTT-Q23-transduced groups. These results collectively demonstrated that mHTT overexpression can promote neuronal apoptosis and insulin signaling blockade.

### 2.3. Liraglutide Alleviates mHTT-Impaired Insulin Downstream Signaling and Oxidative Stress

**Figure 3:**
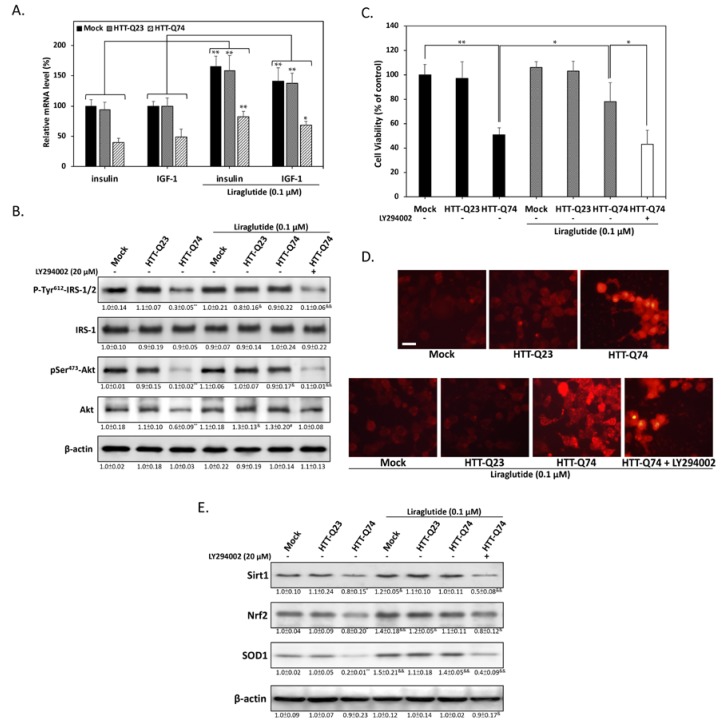
Liraglutide protects against HTT-Q74-induced cell death by restoring insulin signaling. (**A**) Cells were treated for 48 h with or without 0.1 μM liraglutide. The mRNA levels of insulin and IGF-1 were measured through real-time qPCR; (**B**) Immunoblotting revealed that the phosphorylation of Tyr^612^-IRS-1/2 and Ser^473^-Akt was inhibited by 48 h of HTT-Q74 overexpression, and this inhibition was effectively restored by liraglutide; (**C**) Cell viability was determined through MTT assay. The neuroprotective effects of liraglutide were abolished by cotreatment with LY294002 (20 µM), a specific inhibitor of PI3-kinase; (**D**) Dihydroethidium staining results viewed under fluorescence microscopy and showing that liraglutide reduces HTT-Q74-induced intracellular ROS accumulation; (**E**) Levels of some antioxidant signaling-related proteins, including Sirt1, Nrf2, and SOD1, analyzed through Western blot analysis. Liraglutide treatment effectively restored the inhibitory effects of this antioxidant pathway. At least three independent experiments were performed, and values are presented as mean ± SEM. Significant differences were determined through multiple comparisons with Dunnett’s posthoc test for * *p* < 0.05 and ** *p* < 0.01 compared with mock groups, and ^&^ *p* < 0.05 and ^&&^ *p* < 0.01 compared with HTT-Q74 overexpression groups. Scale bar represents 10 μm.

Previous studies have suggested that impaired insulin signaling is a main factor of the pathogenesis of HD [19]. Given that GLP-1 is best known for its ability to facilitate insulin signaling, we next investigated whether liraglutide, a GLP-1 analogue approved and widely used in the treatment of type-2 diabetes, protects against mHTT-induced neurotoxicity. To determine the effects of liraglutide on insulin/IGF-1 gene expression, we performed qPCR assays to analyze the fold change in mRNA levels. As shown in Figure 3A, insulin and IGF-1 mRNA expression increased in cells overexpressing HTT-Q23 and HTT-Q74 treated with liraglutide for 48 h. this result indicated that liraglutide may exert its pharmacological action by stimulating insulin-related downstream signaling in neuronal cells. To determine whether liraglutide can enhance the action of insulin and participates in improving insulin sensitivity, we conducted Western blot analysis to detect the levels of IRS-1/2 phosphorylation at Tyr^612^. As shown in Figure 3B, 48 h of liraglutide treatment restored Tyr^612^ IRS-1/2 phosphorylation. Accordingly, liraglutide also returned the phosphorylation levels of its downstream target Akt Ser^473^ to basal levels, showing that neuronal insulin signaling can be activated by liraglutide during mHTT overexpression. To further investigate the role of liraglutide-mediated insulin signaling, we used the PI3-kinase inhibitor LY294002 as a negative control. LY294002 significantly blocked liraglutide-restored IRS/Akt activation. This result was also confirmed through MTT assays, showing that liraglutide effectively alleviated HTT-Q74-induced cytotoxicity, whereas cotreatment with LY294002 blocked this protective effect (Figure 3C). These results indicated that liraglutide may reduce mHTT-induced cytotoxicity by restoring blocked neuronal insulin signaling. mHTT-induced oxidative stress plays a key role in mHTT-induced neuronal apoptosis [20]. To determine whether liraglutide protects cells from mHTT-induced oxidative stress damage, we measured intracellular ROS levels through the DHE fluorometric method. As expected, our results showed that liraglutide suppressed HTT-Q74-induced ROS intracellular accumulation (Figure 3D). Our Western blot analysis results for Nrf2/HO-1 downstream protein expression also provided further evidence that liraglutide treatment restored the levels of SOD1, a ROS detoxifying enzyme, in HTT-Q74-overexpressing cells (Figure 3E). Taken together, these results imply that mHTT-induced oxidative stress is related to impaired neuronal insulin signaling and is a deleterious factor that increases neurotoxicity.

### 2.4. Liraglutide Increases Autophagy and Alleviates Intracellular HTT Aggregation in HTT-Q74-Overexpressing Cells

**Figure 4:**
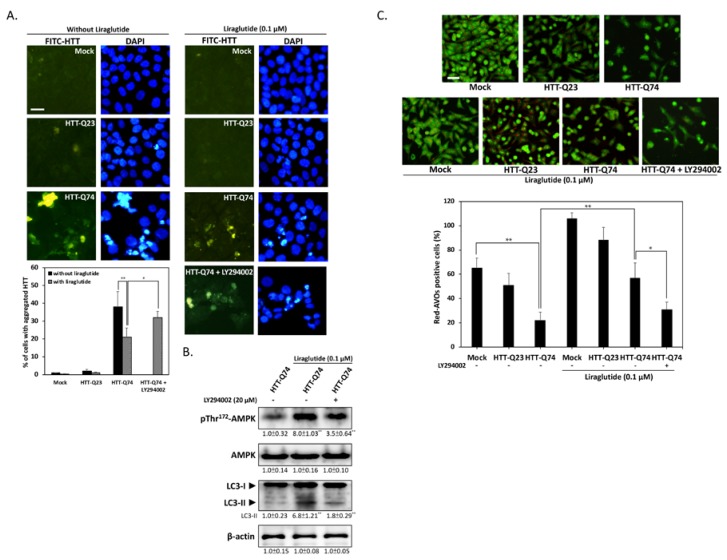
Liraglutide upregulates autophagy by activating AMPK and reducing HTT aggregation in HTT-Q74-overexpressed cells. (**A**) Immunofluorescence images showing that 48 h of HTT-Q74 overexpression increased the number of HTT aggregates. The percentage of cells contained HTT aggregates was calculated from five images of each treatment. Green: HTT, FITC, Blue: nuclei, DAPI; (**B**) Levels of p-AMPK/AMPK and LC3-I/II protein in HTT-Q74-overexpressing cells. Liraglutide markedly upregulated LC3-II and AMPK Thr^172^ phosphorylation. This effect, however, was abolished by LY294002; (**C**) Representative images of AVO-positive cells treated through AO staining. The percentage of red-AVO positive cells was calculated from five images of each treatment. All data were collected from at least three independent experiments, and values are presented as mean ± SEM. The significance of differences was determined through multiple comparisons with Dunnett’s posthoc test for * *p* < 0.05 and ** *p* < 0.01 compared with mock groups. Scale bar represents 20 μm.

Given that mHTT aggregates may have a central role in HD pathogenesis, we investigated whether liraglutide treatment can attenuate HTT aggregation. As shown in Figure 4A, immunofluorescence results revealed that 48 h of HTT-Q74 overexpression significantly stimulated intracellular HTT aggregation. However, liraglutide treatment decreased the number of fluorescent aggregates, and this effect was blocked by LY294002 cotreatment. This indicates that insulin signaling may mediate the beneficial effects of liraglutide by suppressing HTT aggregation in HTT-Q74-overexpressing cells. Autophagy may have a pivotal role in the clearance of intracellular misfolded proteins, such as HTT aggregates [21]. Hence, to identify the molecular basis of liraglutide-mediated neuroprotection, we subjected autophagy-related proteins to Western blot analysis. The results presented in Figure 4B show that liraglutide markedly upregulated the phosphorylation of Thr^172^-AMPK and LC3-II, two positive regulators in autophagy stimulation. Conversely, LY294002 cotreatment significantly blocked this upregulation, indicating that liraglutide-mediated autophagy is dependent on insulin signaling transduction. The results of AO staining also provided further evidence that HTT-Q74 overexpression significantly decreased the number of acidic vesicular organells (AVOs), a marker of autophagy. However, treatment with liraglutide significantly increased the percentage of cells containing AVOs from 22% ± 6.8% to 57% ± 12.4% (Figure 4C). By contrast, cotreatment with LY294002 significantly abolished liraglutide-mediated effects, indicating that the upregulation of autophagy may help attenuate the accumulation of HTT aggregates and thereby protect neuronal cells from mHTT cytotoxicity.

## 3. Discussion

The accumulation of misfolded mHTT proteins in neuronal cells is a central mechanism in HD pathogenesis. Protein clearance is primarily mediated by the ubiquitin–proteasome system (UPS) and the autophagic process [22]. Soluble protein is degraded by the UPS, and protein aggregates that are too large to enter UPS pores undergo autophagy [23]. Thus, the degradation of intracellular toxic aggregates, such as mHTT, is essentially mediated by autophagy [24]. As a result, the activation of the autophagy pathway may help attenuate mHTT toxicity and improve cell viability. In addition, mHTT aggregation can increase cell oxidative stress, suggesting that the aggregates themselves contribute directly to the cytotoxic mechanism of mHTT [25]. Our present observations suggest that mHTT overexpression leads to impaired insulin signaling and HTT aggregation. Conversely, treatment with the GLP-1 analogue liraglutide significantly improves insulin sensitivity and enhances cell viability in mHTT-overexpressing neuronal cells. Furthermore, liraglutide upregulates the expression of genes that encode for endogenous antioxidant enzymes and thus contributes to reductions in oxidative stress and to the attenuation of mHTT-induced neurotoxicity. All these findings indicate that restored insulin signaling may have a protective role in mHTT-overexpressing neuronal cells. A retrospective epidemiologic study showed for the first time that the prevalence of type-2 diabetes in patients with HD is higher than that in normal controls [26]. The risk of HD patients for developing diabetes is 7-fold higher than that of their matched non-HD controls. Most patients with HD and diabetes are under 50 years of age, implying that mHTT may be part of the pathogenesis of type-2 diabetes. The association between HD and diabetes raises the possibility that mHTT-induced insulin resistance may enhance the development of HTT aggregation and neurotoxicity through a mechanism that remains unclear. Given these growing links between insulin resistance and mHTT pathology, the disruption of neuronal insulin signaling in mHTT-overexpressing cells unsurprisingly exacerbates mHTT neurotoxicity. This relationship suggests that approaches that improve insulin signaling, such as treatment with the GLP-1 agonist liraglutide, may be a novel approach for the treatment of HD.

Autophagy has been linked to the pathogenesis of most neurodegenerative disorders, including Alzheimer’s disease, Parkinson’s disease, and HD. Autophagy can function as a survival mechanism by removing abnormal protein aggregates that have accumulated in the cytoplasm and by preserving neuronal function [27,28]. The pharmacological activation of autophagy in animal models have shown strong neuroprotective effects against HD by promoting the clearance of mHTT aggregates from neurons [29]. This indicates that the stimulation of autophagy by liraglutide may be a viable therapeutic solution for attenuating mHTT toxicity. Notably, insulin signaling is traditionally thought to cause the inhibition of autophagy. Thus, the upregulation of autophagy by insulin signaling seems to be self-contradictory. The difference between basal autophagy and stress-induced autophagy may account for this paradox. Basal autophagy is mediated mainly by mTOR signaling, which promotes autophagy to enable cell survival during starvation [30]. Conversely, stress-mediated autophagy can be activated by AMPK via mTOR-independent mechanisms [31]. This phenomenon suggests that insulin signaling may restore autophagy under insulin-resistant stress states, such as mHTT accumulation. Most studies have shown that autophagy deficits may occur during the early stage of AD [32]. AD is also often accompanied by brain insulin resistance. Thus, increasing neuronal insulin sensitivity may help restore autophagy. In parallel with this finding, we previously demonstrated that the restoration of neuronal insulin sensitivity by the DPP4 inhibitor activates AMPK and enhances autophagy-based neuroprotection [16]. Similarly, Panagaki et al. also reported that liraglutide can also elicit ER proteostasis and autophagy activity under chronic stress conditions in neuronal cells [33]. Although our results can be partially rationalized as explained above, the precise mechanism of mHTT-induced insulin signaling blockade requires further elucidation.

In fact, recent evidence suggests that liraglutide may exert beneficial effects on HD progression in a HD transgenic mouse model on glucose homeostasis and insulin resistance [34]. However, the detailed molecular mechanism underling mHTT-associated neuronal insulin resistance remains unclear. In our study, we demonstrated that under mHTT overexpression, insulin signaling upregulates the Sirt1/Nrf2/SOD1 endogenous antioxidant pathway, which drives the removal of HTT aggregates. Interestingly, stress conditions, such as oxidative stress, may promote protein aggregation by lowering the threshold of protein aggregation [35]. Thus, under oxidative stress, aggregation-prone proteins, such as mHTT, that are normally unsusceptible to aggregation become prone to aggregation. This phenomenon indicates that scavenging and decreasing ROS can prevent mHTT toxicity. In fact, increased insulin signaling has been linked to the activation of Sirt1 and Nrf2 [36,37]. Sirt1/Nrf2 activation can counteract oxidative stress by inducing the expression of enzymes, such as SOD, that directly detoxify ROS. This phenomenon suggests that liraglutide may prevent mHTT formation by decreasing ROS levels and modulating endogenous antioxidant pathways through the restoration of insulin sensitivity. Collectively, in the present study, we provided evidence for the ability of liraglutide to inhibit mHTT-induced neurotoxicity. This protective effect appears to be associated with insulin signaling-dependent autophagy activation and Sirt1/Nrf2-related antioxidant pathways. To our knowledge, we are the first to demonstrate the molecular mechanism of liraglutide against mHTT-induced insulin signaling impairment and oxidative damage. Our results provide new insights on the potential use of incretin-based agents, such as liraglutide, in the treatment of HD.

## 4. Materials and Methods

### 4.1. Materials

We purchased chemicals, such as 3-(4,5-dimethylthiazol-2-yl)-2,5-diphenyltetrazolium bromide (MTT), 4′,6-diamidino-2-phenylindole (DAPI), acridine orange (AO), dihydroethidium (DHE), and LY294002, from Sigma (München, Germany). We purchased pEGFP-Q23 HTT (Plasmid# 40261) and pEGFP-Q74 HTT (Plasmid# 40262) from Addgene (Cambridge, MA, USA). We purchased antibodies against caspase 3, poly(ADP-ribose) polymerase (PARP), Akt, p-Akt, AMPK, p-AMPK, Nrf2, and p-IRS-1/2 (Tyr 612) from Santa Cruz Biotechnology (Santa Cruz, CA, USA). Antibodies against IRS-1 were obtained from Cell Signaling Technology (Danvers, MA, USA), and β-actin and LC3 antibodies were obtained from Novus Biologicals (Littleton, CO, USA). Antibodies against SOD1, Sirt1 were purchased from GeneTex (Irvine, CA, USA). Liraglutide was purchased from Novo Nordisk (Copenhagen, Denmark). All chemicals dissolved in phosphate buffer saline solutions and stored at −20 °C until needed for use in experiments.

### 4.2. Cell Culture, Transfection, and MTT Assay

Human neuroblastoma SK-N-MC cells obtained from the American Type Culture Collection (Bethesda, MD, USA) were maintained in Minimal Eagle’s medium (MEM; Gibco) with 10% fetal calf serum, 100 units/mL penicillin, 100 µg/mL streptomycin, and 2 mM l-glutamine; and kept at 37 °C in a humidified atmosphere of 5% CO_2_. Cells were cultured in six-well plates and were transfected after 24 h of culture with 2 μg of pEGFP HTT (Q23)/mHTT (Q74) plasmid or an empty control vector with 5 μL of Lipofectamine 2000 (Life Technologies). Transfections and media changes were performed in accordance with the manufacturers’ optimized protocols. Transfection efficiency at 24 h posttransfection was assessed under fluorescence microscopy on the basis of enhanced green fluorescence gene (EGFP) expression. After 48 h of transfection, cells were treated under the indicated conditions. For MTT viability tests, cells were seeded in 24-well plates overnight and then treated under the indicated conditions. After treatment, MTT was added to the medium in accordance with the manufacturer’s instructions. Only viable cells can metabolize MTT into a purple formazan product, the optical density of which was quantified by a Jasco V-700 spectrophotometer (JASCO, Tokyo, Japan) at 550 nm. The average amount of control cells was set to 100% to enable the comparison of the survival rates of other tested cells.

### 4.3. Microscopic Observation and Acridine Orange–Propidium Iodide Assay

Cells grown on coverslips were examined under microscopy for changes in nuclear morphology characteristic of apoptosis. The cells were fixed in 4% paraformaldehyde after 24 h of treatment with the indicated compounds. Cell images were acquired through phase-contrast inverted microscopy without the use of a specific staining procedure. We utilized a commercial acridine orange (AO)–propidium iodide (PI) staining kit (Logos Biosystems, Annandale, VA, USA) to determine cell viability. AO permeates live and dead cells and generates green fluorescence. However, PI can only enter and stain dead cells. Once bound to nucleic acids, PI fluorescence increases by 20–30-fold and causes the cell to emit red fluorescence. For the AO–PI assay, cells were trypsinized and suspended after treatment under the indicated conditions. Afterward, 2 μL of staining solution was mixed with 18 μL of cell sample and then directly added to cell samples for cell counting and viability analysis on a Luna-FL automated dual fluorescence cell counter (Logos Biosystems) following the manufacturer’s protocol. PI-stained apoptotic cells were quantified by averaging the cell counts of five independent samples. Values were expressed as the percentage of dead cells relative to the total number of cells.

### 4.4. Assessment of Nuclear Morphology through DAPI Staining

Changes in cell nuclear morphology characteristic of apoptosis were examined under fluorescence microscopy. After 24 h treatment with the indicated compounds, cells were fixed in 4% paraformaldehyde, permeabilized in ice-cold methanol, incubated for 15 min with 1 ng/mL DAPI stain at room temperature, and then observed under a fluorescence microscope (DP80/BX53, Olympus, Tokyo, Japan). Apoptotic cells were quantified by counting five random fields per treatment.

### 4.5. Western Blot Analysis

Cells were harvested and lysed with a protein extraction lysis buffer containing 50 mM Tris-HCl (pH 8.0), 5 mM ethylenediaminetetraacetic acid (EDTA), 150 mM sodium chloride (NaCl), 0.5% Nonidet P-40, 0.5 mM dithiothreitol (DTT), 1 mM phenylmethylsulfonyl fluoride (PMSF), 0.15 units/mL aprotinin, 5 μg/ml leupeptin, 1 μg/mL pepstatin, and 1 mM sodium fluoride (NaF). Subsequently, cell debris were removed by centrifugation (12,000× g, 30 min and 4 °C). The supernatant was transferred and used for western blotting analysis. Equal amounts (50 μg) of total proteins were loaded for each well and separated on 10% SDS–PAGE followed by transfer to a polyvinylidene difluoride (PVDF) membrane (Millipore, Bedford, MA, USA). After blocking, the membranes were probed with a primary antibody (1:1000) at 4 °C overnight, and then incubated with appropriate HRP-conjugated secondary antibody (1:5000). Blots were visualized by using the ECL kit (Millipore) with manufacturer's protocol. Immunocomplexes were visualized using enhanced chemiluminescence kits (Millipore). The relative expression levels of proteins were densitometrically quantified using ImagePro Plus 6.0 software (Media Cybernetics, Silver Spring, MD, USA), further normalized on the basis of the expression level of the housekeeping protein β-actin, and then compared with the normalized protein levels of control cells. The control protein level was then set to 100% for comparison. 

### 4.6. mRNA Expression Analysis through Reverse-Transcription Quantitative PCR

We used RNeasy Kit (Qiagen, Germantown, MD, USA) to extract total RNAs and quantified by spectrophotometry. The samples were then added to a reverse transcription mix in a TProfessional Thermocycler (Göttingen, Germany) for converting mRNA to cDNA. The conditions consisted of 25 °C for 10 min followed by 37 °C for 120 min for reverse transcription, and 85 °C for 5 min for reverse transcriptase denaturation. cDNA transcripts were then quantified by reverse-transcription PCR with the ABI 7300 Sequence Detection System (Applied Biosystems, Foster City, CA, USA). 10 ng of cDNA were amplified in triplicate by using Power SYBR Green PCR Master Mix (Applied Biosystems). PCR conditions: initial denaturation at 95 °C for 10 min, followed by 40 two-step cycles—95 °C 15 s, 60 °C 1 min, and finally dissociated at 95 °C 15 s, 60 °C 15 s, 95 °C 15 s. To determine relative mRNA expression, we used the following primer pairs: forward 5′-ACA CCT GTG CGG CTC ACA-3′ and reverse 5′-TCC CGG CGG GTC TTG-3′ for insulin; forward 5′-TGC TTC CGG AGC TGT GAT CT-3′ and reverse 5′- CGG ACA GAG CGA GCT GAC TT-3′ for IGF-1; forward 5′-ATT GCT TGG CTG GTG AAA GG-3′ and reverse 5′-TGT CTG CGG CCA AGT TCT TC-3′ for proglucagon; and forward 5′-TGG TAT CGT GGA AGG ACT CAT GAC-3′ and reverse 5′-ATG CCA GTG AGC TTC CCG TTC AGC-3′ for GAPDH. The values of relative mRNA expression were obtained by using Sequence Detection Systems software (Sequence Detection Systems 1.2.3-7300 Real-Time PCR System; Applied Biosystems) and standardized through comparison with those for the relative expression of GAPDH.

### 4.7. Measurement of ROS thorough Dihydroethidium Staining

To avoid signal interference by the EGFP plasmid, ROS levels were measured through dihydroethidium (DHE) staining. DHE is a cell-permeable fluorogenic probe that reacts with superoxide to form ethidium and emits red fluorescence. After treatment under the indicated conditions, the cells were treated in fresh media containing 10 µM DHE and incubated for 30 min in the dark at room temperature. After incubation, the staining medium was discarded, and the cells were washed twice with PBS. Cell imaging was then performed by using an inverted fluorescence microscope (DP72/CKX41, Olympus).

### 4.8. Immunocytochemistry and AO Staining

After treatment, cells were fixed with 2% buffered paraformaldehyde, permeabilized in 0.25% Triton X-100 (Sigma-Aldrich, München, Germany) for 5 min at 4 °C, and then incubated with anti-αSyn primary antibody. Slides were then incubated with an FITC-labeled second antibody (Santa Cruz) corresponding to primary antibody. Cells were stained with 1 μg/mL AO for 15 min and then washed with MEM medium. Then, images were acquired by using a fluorescence microscope (DP80/BX53, Olympus) and cellSense V 1.9 digital imaging software. The percentage of cells positive for red acidic vesicular organelle (AVO) was calculated from five images of each treatment.

### 4.9. Statistical Analysis

Results are presented as means ± standard error of the means (±SEM). Statistical analysis was performed with use of variance, followed by Dunnett’s posthoc test for multiple comparisons by using SPSS v22.0 statistical software (SPSS, Inc., Chicago, IL, USA). Differences were considered statistically significant wherein * *p* < 0.05 and ** *p* < 0.01 respectively depending on the individual experiments.
